# Vitamin D Status and Components of Metabolic Syndrome in Older Subjects from Northern Finland (Latitude 65°North)

**DOI:** 10.3390/nu11061229

**Published:** 2019-05-30

**Authors:** Shivaprakash Jagalur Mutt, Jari Jokelainen, Sylvain Sebert, Juha Auvinen, Marjo-Riitta Järvelin, Sirkka Keinänen-Kiukaanniemi, Karl-Heinz Herzig

**Affiliations:** 1 Research Unit of Biomedicine, Department of Physiology, University of Oulu, 90014 Oulu, Finland; 2 Medical Research Center (MRC), University of Oulu, 90014 Oulu, Finland; Juha.Auvinen@oulu.fi; 3 Biocenter of Oulu, University of Oulu, 90014 Oulu, Finland; Sylvain.Sebert@oulu.fi (S.S.); m.jarvelin@imperial.ac.uk (M.-R.J.); 4 Center for Life Course Health Research, Faculty of Medicine, University of Oulu, 90014 Oulu, Finland; Jari.Jokelainen@oulu.fi (J.J.); sirkka.keinanen-kiukaanniemi@oulu.fi (S.K.-K.); 5 Unit of General Practice, Oulu University Hospital, 90220 Oulu, Finland; 6 Department of Genomics of Complex Diseases, School of Public Health, Imperial College London, London W2 1PG, UK; 7 Institute of Health Sciences, University of Oulu, 90014 Oulu, Finland; 8 Unit of Primary Care, Oulu University Hospital, 90220 Oulu, Finland; 9 Department of Children, Young People and Families, National Institute for Health and Welfare, 90101 Oulu, Finland; 10 Department of Epidemiology and Biostatistics, and MRC-PHE Center for Environment and Health, School of Public Health, Imperial College London, London W2 1PG, UK; 11 Department of Gastroenterology and Metabolism, Poznan University of Medical Sciences, 61-701 Poznan, Poland

**Keywords:** aging, vitamin D status, 25OHD, vitamin D supplementation, insulin resistance, HOMA, HDL cholesterol, LDL cholesterol, blood pressure, metabolic syndrome

## Abstract

Introduction: Vitamin D deficiency has been linked to the increased risk of several chronic diseases, especially in people living in the Northern Latitudes. The aim of this study was to assess the vitamin D status in older subjects born in 1945 in Northern Finland (latitude 65°North), and to examine its associations to components of metabolic syndrome (MetS). Methods: In this cross-sectional study, we invited 904 subjects born in 1945 from the Oulu region (Oulu45 cohort), out of an original cohort of 1332 subjects. In the cohort, plasma 25 hydroxyvitamin D (25OHD) levels were determined by an enzyme immunoassay of 263 men and 373 women, with a mean age baseline of 69±0.5 years old. We assessed the participants’ usage of vitamin D supplements, as well as their lifestyle factors, using a questionnaire. Results: Nearly 80% of the subjects had low vitamin D levels [either vitamin D deficient (<50 nmol/L) or insufficient (50 – 75 nmol/L)], and only 20% of the participants had sufficient vitamin D levels (>75 nmol/L) (based on the American Endocrine Society guidelines). The low vitamin D status was associated with a high prevalence of MetS; a significantly higher number of subjects with MetS (41%) had low vitamin D levels in comparison to the non-MetS subjects (38%) (*p* ≤ 0.05). The subjects under vitamin D supplementation had a significantly lower incidence of MetS (42.6% vs 57.4%) and its components in comparison to the non-supplemented subjects (*p* ≤ 0.05). Conclusions: Low vitamin D levels are a risk factor for MetS amongst other lifestyle factors, such as dietary habits and physical inactivity, among older subjects in the Northern Latitudes (65°North). Optimal supplementation of vitamin D, along with rich dietary sources of vitamin D, are highly recommended for older subjects as a means to positively affect, e.g., hypertension, insulin resistance, and obesity, as components of the MetS.

## 1. Introduction 

Vitamin D deficiency is a pandemic [1,2] and particularly common amongst older people living in the Northern latitudes. The deficiency results from insufficient solar radiations for nearly half the year [3,4] and reduced cutaneous synthesis during aging [5]. Vitamin D deficiency is primarily associated with poor musculoskeletal health, osteoporosis, and osteomalecia [3]. Observational studies demonstrate that low circulating 25 hydroxyvitamin D levels (25OHD, inactive metabolite and maker of vitamin D status) could be implicated in the etiology of several chronic diseases, such as type 2 diabetes (T2D), cardiovascular disorder (CVD), obesity, and cancer, as well as all-cause mortality [6,7,8,9,10].

Metabolic syndrome (MetS) comprises increased fasting plasma glucose (FPG), abdominal obesity, high triglyceride (TG) levels, reduced high-density lipoprotein (HDL) cholesterol, and high blood pressure (BP) [11]. The increased MetS prevalence may be explained by aging societies and the increase in adiposity, due to low physical activity and habitual lifestyle factors. Excess adiposity contributes to low-grade systemic inflammation and impairs metabolic functions [12,13]. Although there are no unifying pathways to sufficiently explain the pathogenesis of MetS, the most common denominator is adiposity with an increased subacute inflammatory load, thereby contributing to insulin resistance and dyslipidemia [13]. Previously, we have shown that vitamin D attenuated the activation of the proinflammatory transcription factor nuclear factor kappa-beta (NFκB) in adipocytes [14]. Several epidemiological studies suggest that lower 25OHD concentrations are associated with a higher prevalence of MetS [15,16,17,18,19], whilst other studies did not find any association [20,21]. These inconsistent reports might be due to variations in the study populations, such as participant age, geographical location, life style factors, regional dietary and seasonal differences, food fortification, and the use of vitamin D supplements. Although dietary recommendations, including food fortification, are implemented in many European countries [22], recent studies have reported that the dietary supplementations of vitamin D are insufficient to provide adequate vitamin D levels in adolescent subjects [23]. 

In 2003 and 2010, Finland implemented initial nutrition policy actions on vitamin D intake via food fortification, wherein the food fortification with vitamin D was doubled [24,25,26]. However, no community based study report is currently available regarding the usage of vitamin D supplements, and the association between circulating 25OHD and MetS and its components, amongst older subjects living in Northern Finland. Therefore, we sought to investigate the associations between serum 25OHD levels and the prevalence of MetS and its components. Furthermore, we assessed the effects of vitamin D supplementation on MetS.

## 2. Methods

### 2.1. Study Population

**Figure 1:**
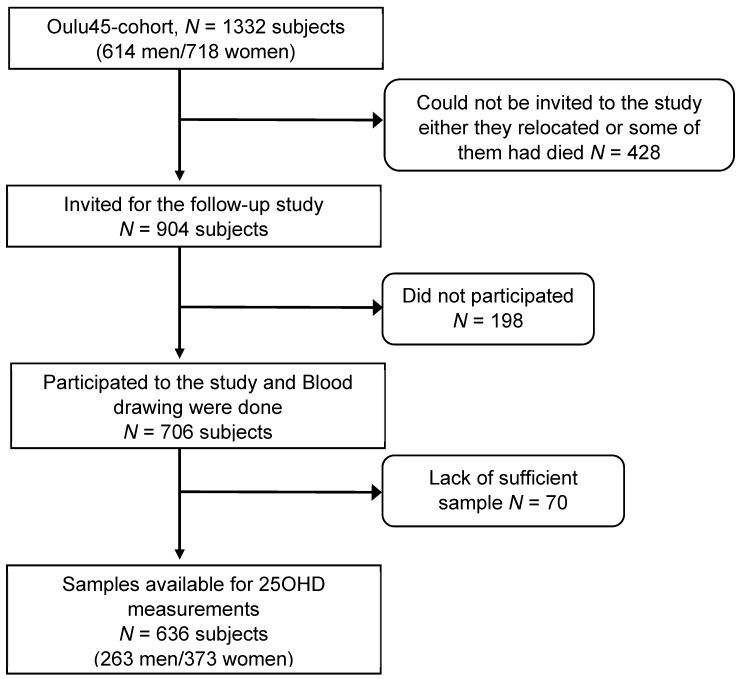
Flow chart of the study.

In 2001, all subjects born in 1945 in the city of Oulu, Finland (Oulu-45 cohort, latitude 65°N), and living in Oulu, were invited to participate in a data collection that was carried out from 2001–2002. In 2013, 904 subjects that were alive and living in Oulu, were invited for a follow-up study from 2013–2015 (Figure 1). From the participants, 198 subjects declined to participate in the clinical examinations, and in 70, there was a lack of sufficient serum samples for all the analyses. The ethical committee of the Northern Ostrobothnia Hospital District approved this study in compliance with the National Guidelines and legislation, as well as the Declaration of Helsinki. All the participants gave their written informed consent to participate. The study protocol for anthropometric measurements and clinical data collection are described in detail previously [27].

### 2.2. Study Protocol

After an overnight fast, blood samples were drawn from the participants to establish fasting parameters, and an oral glucose tolerance test (OGTT) was performed. Participants were diagnosed as T2D patients if either they had been diagnosed earlier, were taking diabetes medication, had a fasting blood glucose (FBG) value ≥7.0 mmol/L, or the 2-hour OGTT blood glucose (2h BG) value ≥11.1 mmol/L. Participants were considered to have an impaired fasting glucose (IFG) if they had increased fasting blood glucose (6.1–6.9 mmol/L). They were considered to have an impaired glucose tolerance (IGT) if they showed an increased 2h BG (7.8–11.1 mmol/L). All the other participants were regarded as normal glucose tolerance (NGT) subjects. Blood was drawn between May and October, which was considered as the summer season, and between November and April, which was the winter season, based on changes in the availability of solar radiations (Oulu 65° north) for cutaneous vitamin D synthesis [4]. 

The subjects underwent bioelectrical impedance measurements (InBody 720, InBody, Seoul, Korea). In all the participants, the habitual physical activity was objectively measured using a wrist-worn acceleration meter (Polar Active, Polar Electro, Finland) as previously described [28]. The participants filled in questionnaires on vitamin D supplementation along with life style factors, such as smoking habits, alcohol consumption, mental status, and perceived health. Based on the questionnaires, the subjects who regularly or occasionally used vitamin D supplements were categorized as vitamin D supplemented. 

### 2.3. 25OHD Measurements

Serum 25OHD levels were measured using a 25OHD enzyme immunoassay (EIA) kit (Immunodiagnostic Systems GmbH, Germany) following the manufacturer’s instructions. As reported previously, for accuracy assessments, each measurement was included with certified material from the National Institute of Standards and Technology, United States (NIST, USA); Standard Reference Material-972 (SRM-972; level 1) and internal plasma controls [29]. 

The inter-assay variations (CVs) ranged between 6.2–9.3% for the controls and certified material. All the measurements were performed blinded to the clinical data.

### 2.4. Definitions of MetS and Vitamin D Status

MetS was defined using the International Diabetes Federation (IDF) definition with the following criteria: waist circumference (WC) ≥94 cm for men and ≥80 cm for women, TG ≥150 mg/dL, HDL cholesterol ≤40 mg/dL for men and ≤50 mg/dL for women, blood pressure ≥130 / ≥85 mmHg, and fasting glucose ≥100 mg/dL. Participants were diagnosed to have MetS if they exhibited central adiposity in addition to two of the listed risk factors [11].

Vitamin D status was divided into three categories based on the definition of the American Endocrine Society [30]: 25OHD levels <50 nmol/L represented vitamin D deficiency, 25OHD levels between 50 nmol/L to 75 nmol/L represented vitamin D insufficiency, and 25OHD levels >75 nmol/L were considered as vitamin D sufficient [31].

In addition, in 2013, the European Society for Clinical and Economic Aspects of Osteoporosis and Osteoarthritis (ESCEO) suggested the use of 50 nmol/L as the minimal serum 25OHD concentration in the elderly population, as well as in patients with osteoporosis to ensure optimal bone health. Below this threshold, they recommended supplementation with 800 to 1000 IU/day [32].

### 2.5. Statistics

The 25OHD levels (nmol/L) were mean ± SD for the continuous variables, and percentages for the categorical variables. P-value of <0.05 was considered significant. The Kruskal–Wallis test was used for comparisons of the continuous variables between groups. ANCOVA was used to compare the vitamin D levels amongst the participants, adjusted for BMI and lifestyle factors. Differences in the frequency were tested using the chi-square test. The Spearman’s rho was used to compare the continuous variables. Multiple regression analysis was performed to assess the linear associations between vitamin D levels and MetS, before and after, adjustments for the season of sampling, BMI, lifestyle factors, and gender. In the analysis, there were few missing values for some of the variables (i.e., physical activity, season of sampling, and smoking status). Statistical analyses were done using R version 3.2.3 and IBM SPSS Statistics 21.

## 3. Results

The vitamin D levels from 636 participants (373 women and 263 men) with a mean age of 69 ± 0.5 years were measured (Figure 1). From the cohort, 20.6% of the subjects were vitamin D sufficient (25OHD levels >75 nmol/L), 57.5% were vitamin D insufficient (25OHD levels 50–75 nmol/L), and 21.9% were vitamin D deficient (25OHD levels <50 nmol/L). The vitamin D levels and baseline characteristics, including lifestyle factors, are presented in Table 1. The vitamin D levels were significantly associated with gender, season of blood sampling, vitamin D supplementation, and physical activity (*p* < 0.05). Smoking and alcohol consumption had no effect on vitamin D levels. The vitamin D levels were associated with the anthropological and biochemical parameters of the study populations (Table 2). Vitamin D levels were inversely associated with BMI, WC, fasting glucose, fasting insulin, total cholesterol, TG levels, and the homeostatic model assessment for insulin resistance (HOMA-IR) (*p* < 0.05). Furthermore, sufficient 25OHD levels were associated with increased HOMA insulin sensitivity (S) scores (HOMA-S 81.8 vs. 70.9 and 64.0, *p* < 0.001) in comparison to the respective deficient and insufficient 25OHD levels. Associations between low vitamin D levels and lipid parameters, such as TG, total, and LDL cholesterol, remained significant (*p* = 0.05) after adjustment for BMI and baseline characteristics, including the lifestyle factors presented in Table 1. 

### Vitamin D Levels and the components of MetS

Amongst the 319 MetS subjects, 26.3% of them were vitamin D deficient and 55.2% were vitamin D insufficient. The low vitamin D levels had significant associations with WC, where 25.8% of the subjects were deficient and 55.6% were insufficient (*p* < 0.001). TG, HDL, and BP were higher in the subjects with insufficient vitamin D compared to the sufficient vitamin D level subjects, although the difference did not reach statistical significance (Table 3). However, FBG (≥110 mg/dl) (*p* = 0.058) showed a weak but insignificant association with low vitamin D. 

**Figure 2:**
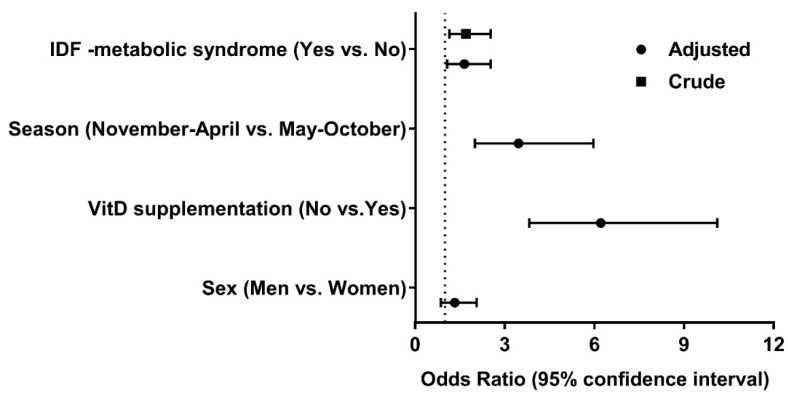
Forest plot for the risk of low levels (less than 75 nmol/L) of Vitamin D. Adjusted odds ratios and their 95% confidence interval for each factor. IDF: International Diabetes Federation, VitD: vitamin D.

Vitamin D supplementation records from our subjects were evaluated in association with the MetS. Of the 319 MetS subjects, 57.4% of the subjects were without vitamin D supplementation compared to 42.6% of the subjects who supplemented their vitamin D levels. Subjects without vitamin D supplementation has significant correlations with one or more components of MetS, except for the TG (*p* = 0.07; Table 3). Thus, the supplementation of vitamin D was associated with a lower incidence of MetS. We also assessed the contributing risk factors for the low circulating vitamin D levels. The forest plot (Figure 2) revealed that a lack of vitamin D supplementation (OR = 6.22; 95% CI, 3.82–10.11; *p* ≤ 0.0001) and the winter season (OR = 3.46; 95% CI, 2.01–5.97; *p* ≤ 0.0001) were important risk factors for low vitamin D levels. Importantly, low vitamin D levels were associated with a higher risk of MetS (OR = 1.70; 95% CI, 1.15–2.53; *p* = 0.008), and the association remained significant even after adjustment for the season of sampling (November-April), vitamin D supplementation, and the gender of the subjects (OR = 1.65; 95% CI, 1.08–2.53; *p* = 0.021). This was in line with our previous study, which demonstrated that a higher BMI leads to lower 25OHD, whilst any effects of lower 25OHD increasing BMI were likely to be minimal [33].

## 4. Discussion

In our cross-sectional cohort (69 years), we found that vitamin D levels were significantly associated with the MetS, and vitamin D supplementation had a beneficial effect on the components of MetS. Our study subjects, living near the Arctic Circle (200 km south), had a prevalence of vitamin D deficiency of 21.9%, whilst 57.5% were insufficient, which was in accordance with previous studies from Finland and Northern Europe [6,26]. 

Vitamin D is primarily produced in the epidermis of the skin from 7-dehydrocholesterol via a photochemical reaction using ultraviolet-B radiation (UVB, wavelength 290–315nm) [5]. The seasonal variation of serum vitamin D levels in Northern Finland (~65 degrees) is obvious due to the changes in luminosity, which is only sufficient during the months of June to October [4]. In addition, vitamin D can be obtained from a person’s diet, such as fatty fish and mushrooms [3]. Although we did not have a detailed food record for our cohort, based on a published dietary survey of 8960 subjects born in 1966 in the same region, roughly one fourth of the cohort had a regular fish intake [34]. Previously, we conducted a Nordic multi-center trial with 213 MetS subjects, which were randomized to control for a healthy Nordic diet, favoring increased fish consumption (≥300 g/week, including ≥200 g/week of fatty fish) amongst other healthy Nordic products [35]. Our healthy Nordic diet intervention did not increase the plasma 25OHD concentration. The reason why fish consumption did not improve the vitamin D levels, might be that farmed fish contains less vitamin D or that frying the fish might have resulted in vitamin D destruction [36]. Fortification of milk products and dietary fats with vitamin D commenced in 2003 [24]. Owing to the low dietary intake of vitamin D and the risk of vitamin D deficiency in Finland [23], the fortification level was increased in 2010. The fortification increased the average vitamin D intake in the Finnish adult population, although in older women (65–74 years), the mean vitamin D intake from food was only 9 µg/day, which was below the vitamin D recommendations [25].

The hypovitaminosis D in our subjects was associated with a significantly higher risk of MetS, abdominal obesity, and higher fasting glucose in comparison to vitamin D sufficient subjects. Previously, we have shown that BMI was causally associated to low vitamin D using the bi-directional Mendelian randomization approach [33]. Increased circulating 25OHD levels have been reported after weight-loss interventions [37]. Adiposity and inflammatory cytokines interfere with the normal metabolism, disrupt insulin signaling, thereby contributing to insulin resistance [38]. Several epidemiological studies reported that lower circulating 25OHD levels were associated with increased metabolic abnormalities, such as inflammation, dyslipidemia, and insulin resistance [6,7]. Our study showed a significant inverse association between 25OHD levels and TG, total cholesterol, and LDL cholesterol. These associations were independent of body weight and life style factors (Table 1). The vitamin D levels were significantly associated with the glycemic markers of insulin resistance, such as fasting glucose, fasting insulin, and HOMA-IR. HOMA-S was significantly increased in the subjects with the sufficient 25OHD levels. However, the associations with glycemic markers became weaker after adjustment for BMI and life style factors. 

The prevalence of MetS in older populations is alarmingly high. Our results were also in agreement with previous reports [15,16,17,18,19]. Vitezova et al. reported that in a cross-sectional cohort from Rotterdam involving 3240 older subjects, the prevalence of MetS lowered with every 10 nmol/L increase in the circulating 25OHD levels [16]. Furthermore, they found that vitamin D levels were significantly associated with a lower prevalence of WC, TG, BP, fasting glucose, and lower HDL-C. Our findings were also in line with the Furukawa study, consisting of 1790 subjects (18–69 years old), which demonstrated that higher vitamin D levels were significantly associated with a lower incidence of MetS in older subjects (≥44 years). However, no significant associations were found with the individual MetS components [19]. Recently, Ju et al showed an inverse association between serum 25OHD concentrations and the risks of MetS in a dose-response meta-analysis study, consisting of 41,471 participants (18–96 years old) from 18 independent observational studies (six cross-sectional studies, one cohort, and one nested case-control study). Significant associations were only found in 16 cross-sectional studies (OR 0.87), but not in the two longitudinal studies (OR 1.00). Furthermore, the authors concluded that the associations were stronger in the older populations from geographical locations that were north of the 38 °N latitude, and in populations with MetS [39]. 

It has long been recognized that aging decreases the circulating vitamin D levels and increases the risk of several metabolic disorders. However, a limited number of studies have analyzed the effects of vitamin D supplementation in older subjects with MetS, living at higher latitude. In the present study, we tried to address the associations between vitamin D and MetS by utilizing questionnaire data on vitamin and mineral supplements. Subjects who supplemented themselves had a lower prevalence of MetS. Furthermore, we found that supplementation significantly lowered the risk of vitamin D deficiency in our cross-sectional study (Figure 2). These findings were in agreement with other cross-sectional, longitudinal, and RCT studies with vitamin D supplementation and its associations with MetS [15,40,41,42]. 

The strength of our study is that all participants were from a single region (Oulu) from Northern Finland (latitude 65°North), with the same age, ethnic background, life style, and environment. From our questionnaire data, we were not able to calculate the exact dose of vitamin D supplementation, since our subjects used various vitamin and mineral supplements that were available in the Finnish pharmaceutical market. In addition, we did not have sufficient detailed dietary records to calculate the food specific vitamin D supply. Our data were generated from a cross-sectional cohort of older subjects, and therefore, our associations do not imply the causality. However, the beneficial effects of vitamin D supplementation on hypertension, insulin resistance, and obesity have been documented in several other publications [15,16,17,18,19]. Nevertheless, the effect of vitamin D on MetS still needs further investigation using well-designed supplementation trials.

## 5. Conclusions

In conclusion, sufficient 25OHD levels (>75nmol/L) were inversely associated with the components of MetS in our older cohort. Moreover, insufficient vitamin D levels were strongly associated with increased abdominal obesity and higher fasting blood glucose levels. In addition, subjects with vitamin D supplementations had less components of MetS (obesity, insulin resistance, and hypertension). Our data suggests that older subjects living in Northern Europe stand to benefit from higher serum 25OHD levels, and thus, vitamin D supplementation is highly recommended. 

## Figures and Tables

**Table 1:** Baseline characteristics of the subjects—25OHD levels.

	Total	Deficiency (<50 nmol/L)	Insufficiency (50–75 nmol/L)	Sufficiency (≥75 nmol/L)	*p* Value
Number of subjects	636	139 (21.9)	366 (57.5)	131 (20.6)	
Age (years)	69.0 ± 0.5	68.9 ± 0.5	69.0 ± 0.5	69.0 ± 0.5	0.241
Gender					0.030
Men	263 (41.2)	63 (24.0)	159 (60.5)	41 (15.6)	
Women	373 (58.8)	76 (20.4)	207 (55.5)	90 (24.1)	
Physical activity					<0.001
Sedentary	55 (9.2)	23 (41.8)	29 (52.7)	3 (5.5)	
Moderate	345 (58.0)	79 (22.9)	195 (56.5)	71 (20.6)	
Vigorous	195 (32.8)	32 (16.4)	115 (59.0)	48 (24.6)	
Season of blood sampling					<0.001
May-October	170 (29.5)	20 (11.8)	110 (64.7)	40 (23.5)	
November-April	406 (70.5)	113 (27.8)	223 (54.9)	70 (17.2)	
Vitamin D supplements					<0.001
No	328 (51.6)	111 (33.8)	177 (54.0)	40 (12.2)	
Yes	308 (48.4)	28 (9.1)	189 (61.4)	91 (29.5)	
Smoking					0.090
Current	76 (12.2)	25 (32.9)	40 (52.6)	11 (14.5)	
Former	219 (35.3)	45 (20.5)	120 (54.8)	54 (24.7)	
Never	326 (52.5)	69 (21.2)	192 (58.9)	65 (19.9)	
Alcohol	1.7 ± 3.6	2.5 ± 4.7	1.5 ± 3.3	1.6 ± 2.7	0.336

Values are expressed as either means ± standard deviations or the numbers of subjects and percentages presented in parentheses. *p* values were calculated using the Kruskal–Wallis rank sum test or chi-square test between the groups.

**Table 2:** Baseline anthropological and biochemical characteristics of the subjects by 25OHD status.

	Total	Deficiency (<50 nmol/L)	Insufficiency (50–75 nmol/L)	Sufficiency (≥75 nmol/L)	*p* Value	*p* * Value	*p* ** Value
25OHD levels (nmol/L)	62.9 ± 18.5	41.5 ± 7.1	61.7 ± 6.8	89.1 ± 17.3	<0.001		
BMI	27.6 ± 4.8	28.6 ± 4.8	27.7 ± 4.7	26.4 ± 4.7	<0.001	0.132	
Waist circumference (cm)	93.8 ± 13.7	96.7 ± 14.0	94.0 ± 13.0	90.3 ± 14.3	<0.001	0.286	0.847
Lean body mass (kg)	47.0 ± 9.6	47.6 ± 9.7	47.4 ± 9.7	45.2 ± 9.0	0.143	0.516	0.817
Fat mass (kg)	26.1 ± 10.1	28.0 ± 10.5	26.0 ± 10.4	24.7 ± 8.7	0.083	0.488	0.369
Fasting glucose (mmol/L)	5.7 ± 1.0	5.8 ± 0.8	5.8 ± 1.2	5.6 ± 0.7	0.010	0.502	0.903
Fasting insulin (mmol/L)	15.4 ± 17.3	17.4 ± 18.5	15.7 ± 18.8	12.6 ± 9.4	<0.001	0.225	0.737
Total cholesterol (mmol/L)	5.3 ± 1.2	5.6 ± 1.3	5.2 ± 1.2	5.2 ± 1.3	0.024	0.004	0.001
HDL cholesterol (mmol/L)	1.7 ± 0.5	1.6 ± 0.5	1.6 ± 0.5	1.7 ± 0.5	0.084	0.761	0.369
LDL cholesterol (mmol/L)	3.4 ± 1.1	3.6 ± 1.2	3.3 ± 1.0	3.2 ± 1.1	0.052	0.036	0.021
Triglycerides (mmol/L)	1.3 ± 0.7	1.5 ± 1.2	1.2 ± 0.5	1.0 ± 0.4	<0.001	<0.001	0.001
DP (mmHg)	85.5 ± 9.6	87.0 ± 8.7	85.2 ± 9.9	84.8 ± 9.6	0.057	0.182	0.332
SP (mmHg)	144.1 ± 17.4	144.6 ± 17.2	144.3 ± 16.7	143.2 ± 19.4	0.600	0.242	0.272
HOMA_B	109.4 ± 41.9	115.0 ± 43.6	108.7 ± 41.1	105.3 ± 41.9	0.058	0.301	0.545
HOMA_S	71.7 ± 39.8	64.0 ± 36.1	70.9 ± 39.5	81.8 ± 42.7	<0.001	0.243	0.610
HOMA_IR	1.9 ± 1.3	2.1 ± 1.5	1.9 ± 1.3	1.7 ± 1.2	<0.001	0.106	0.342

Values are expressed as either means ± standard deviations. *p* *: adjusted for the baseline characteristics presented in Table 1. *p* **: *p* *+ BMI adjusted values. *p* value were calculated using the Kruskal-Wallis rank sum test or the chi-square test between the groups; *p* * and *p* ** calculated using ANCOVA. BMI: body mass index, HDL: high-density lipoprotein, LDL: low-density lipoprotein, DP: diastolic blood pressure, SP: systolic blood pressure, HOMA: homeostatic model assessment, IR: insulin resistance, S: insulin sensitivity, B: pancreatic β cell function.

**Table 3:** 25OHD level in relation to metabolic syndrome, waist circumference, triglyceride, HDL cholesterol, blood pressure, and fasting glucose.

Components	Total Cases	25OHD Level						*p* Value	Vitamin D Supplementation				*p* Value
		Deficiency (<50 nmol/L)		Insufficiency (50–75 nmol/L)		Sufficiency (≥75 nmol/L)			No		Yes		
		Number	Percentage	Number	Percentage	Number	Percentage		Number	Percentage	Number	Percentage	
Metabolic syndrome (IDF)													
No	313	54	17.3	187	59.7	72	23.0	0.016	141	45.1	172	54.9	<0.001
Yes	319	84	26.3	176	55.2	59	18.5		183	57.4	136	42.6	
Waist circumference (cm)													
Men < 94 & Women < 80	175	20	11.4	109	62.3	46	26.3	<0.001	83	47.4	92	52.6	0.043
Men ≥ 94 & Women ≥ 80	457	118	25.8	254	55.6	85	18.6		241	52.7	216	47.3	
Triglyceride (mg/dL)													
<150	374	80	21.4	222	59.4	72	19.2	0.491	183	48.9	191	51.1	0.074
≥150	259	58	22.4	144	55.6	57	22.0		144	55.6	115	44.4	
HDL cholesterol (mg/dL)													
Men ≥ 40 & women ≥ 50	394	85	21.6	232	58.9	77	19.5	0.732	193	49.0	201	51.0	0.034
Men < 40 & women<50	239	53	22.2	134	56.1	53	21.7		134	56.1	105	43.9	
Blood pressure (mmHg)													
No (<130/85)	106	19	17.9	58	54.8	29	27.3	0.192	43	40.6	63	59.4	0.006
Yes (≥130/85)	527	120	22.8	305	57.9	102	19.3		282	53.5	245	46.5	
Fasting glucose (mg/dL)													
<100	296	59	19.9	164	55.4	73	24.7	0.058	128	43.2	168	56.8	<0.001
≥100	337	80	23.7	199	59.1	58	17.2		197	58.5	140	41.5	
